# Repositioning CEP-1347, a chemical agent originally developed for the treatment of Parkinson’s disease, as an anti-cancer stem cell drug

**DOI:** 10.18632/oncotarget.22033

**Published:** 2017-10-24

**Authors:** Masashi Okada, Hiroyuki Takeda, Hirotsugu Sakaki, Kenta Kuramoto, Shuhei Suzuki, Tomomi Sanomachi, Keita Togashi, Shizuka Seino, Chifumi Kitanaka

**Affiliations:** ^1^ Department of Molecular Cancer Science, Yamagata University School of Medicine, Yamagata 990-9585, Japan; ^2^ Department of Clinical Oncology, Yamagata University School of Medicine, Yamagata 990-9585, Japan; ^3^ Department of Obstetrics and Gynecology, Yamagata University School of Medicine, Yamagata 990-9585, Japan; ^4^ Department of Ophthalmology, Yamagata University School of Medicine, Yamagata 990-9585, Japan; ^5^ Research Institute for Promotion of Medical Sciences, Yamagata University Faculty of Medicine, Yamagata 990-9585, Japan

**Keywords:** mixed lineage kinase (MLK), c-Jun N-terminal kinase (JNK), serial transplantation assay, xenograft, brain tumor

## Abstract

CEP-1347 is a mixed lineage kinase inhibitor tested in a large-scale phase 2/3 clinical trial in early Parkinson’s disease, in which its safety and tolerability, but nevertheless not efficacy, was demonstrated. Here we identify by drug repositioning CEP-1347 as a potential anti-cancer stem cell drug. *In vitro*, CEP-1347 efficiently induced differentiation and inhibited the self-renewal and tumor-initiating capacities of human cancer stem cells from glioblastoma as well as from pancreatic and ovarian cancers at clinically-relevant concentrations, without impairing the viability of normal fibroblasts and neural stem cells. *In vivo*, a 10-day systemic administration of CEP-1347 at a dose that was less than 1/10 the mouse equivalent of the dose safely given to humans for 2 years was sufficient to effectively reduce tumor-initiating cancer stem cells within established tumors in mice. Furthermore, the same treatment protocol significantly extended the survival of mice receiving orthotopic implantation of glioma stem cells. Together, our findings suggest that CEP-1347 is a promising candidate for cancer stem cell-targeting therapy and that further clinical and preclinical studies are warranted to evaluate its efficacy in cancer treatment.

## INTRODUCTION

Cancer stem cells (CSCs) are a small, specific subpopulation of cancer cells characterized by self-renewal and tumor-initiating capacities, often associated with increased resistance to conventional chemo- and radiotherapies. As such, CSCs survive and repopulate the tumor after seemingly successful treatment, serving as a potential source of post-treatment recurrence and metastasis. Eradication of CSCs, therefore, is now considered key to successful, long-term control of cancer through prevention of recurrence and metastasis [1–3]. Accordingly, drugs targeting CSCs have been actively and enthusiastically pursued, and a plethora of candidates are currently under preclinical and/or clinical development [1]. However, to our knowledge, no drug is yet available and has reached clinical practice for which efficacy against CSCs is demonstrated clinically in humans.

Drug repositioning has emerged as a potential strategy of drug development that offers substantial benefits over *de novo*, traditional drug discovery by reducing development risks as well as by facilitating the development process [4, 5]. Taking advantage of this newer strategy, a number of attempts have been made so far to identify CSC-targeting agents from among existing drugs originally developed for other indications, with promising results including ours [6–10]. Here, we report another instance of such drug repositioning, where we successfully unveiled the anti-CSC activity of CEP-1347, a mixed lineage kinase (MLK) inhibitor which was developed but eventually failed to show its efficacy to treat Parkinson’s disease [11, 12]. Our findings indicate that CEP-1347, being a potent driver of CSC differentiation into non-CSCs *in vitro*, effectively inhibits tumor initiation by CSCs *in vivo*, suggesting that CEP-1347 may be a promising candidate for an anti-CSC agent.

## RESULTS

### CEP-1347 promotes the differentiation of CSCs into non-CSCs *in vitro*

To identify novel CSC-targeting agents by drug repositioning, we screened a panel of candidate existing drugs that were presumed to have CSC-inhibitory activity based on their mechanisms of action, by examining their effect on the expression of stem cell marker expression in CSCs. Among such candidate drugs was CEP-1347, an inhibitor of MLKs that act upstream of c-jun N-terminal kinase (JNK) in the JNK signaling cascade [12]. Since CEP-1347 as such can inhibit the JNK signaling pathway, which has been shown to be essential for the maintenance of a variety of CSCs [7, 13], we examined its effect on human CSCs known to be dependent on JNK.

**Figure 1:**
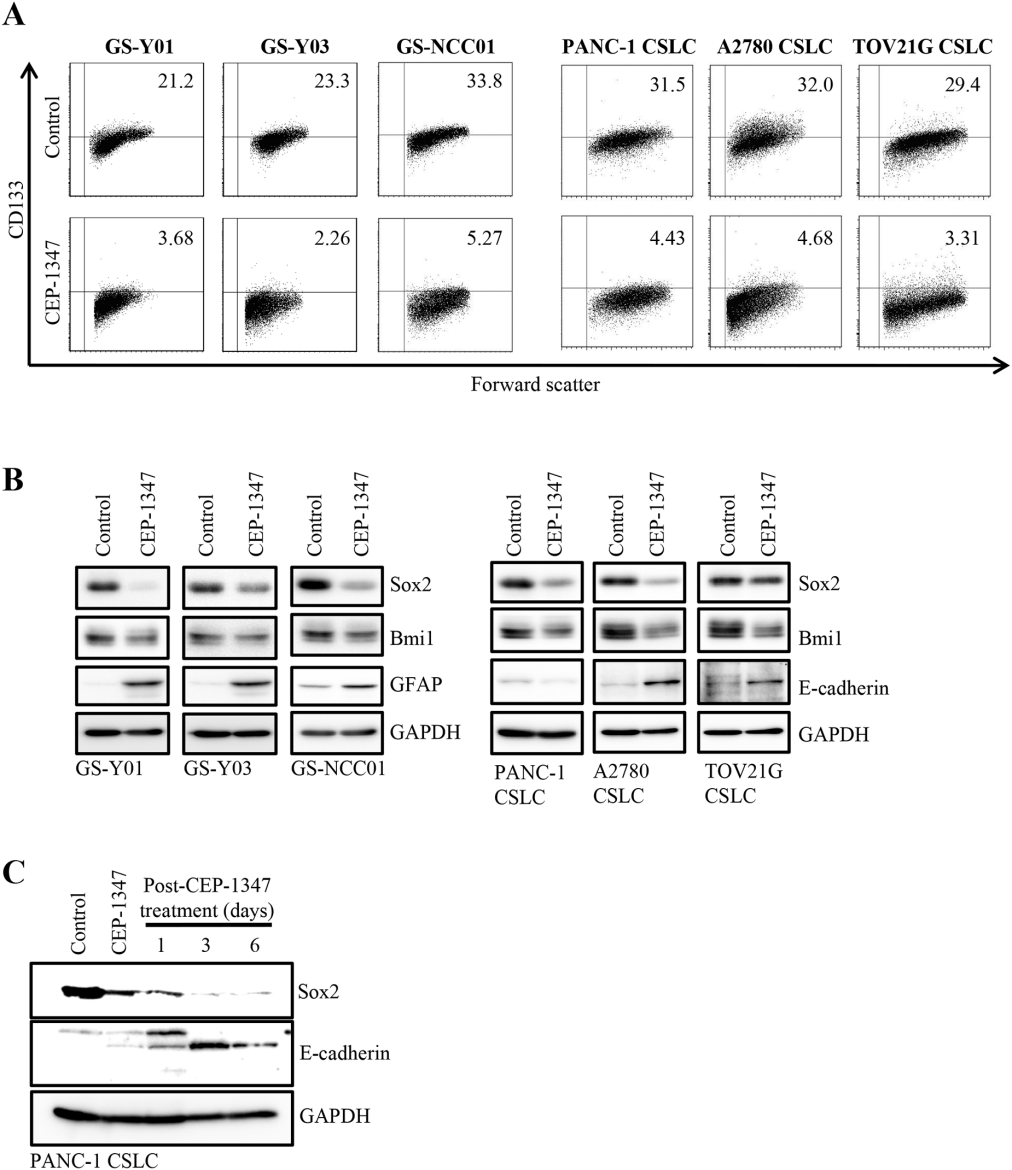
CEP-1347 induces the differentiation of cancer stem cells into non-cancer stem cells **(A)** Glioma stem cells (GS-Y01, GS-Y03, and GS-NCC01), pancreatic cancer stem cells (PANC-1 CSLC), and ovarian cancer stem cells (A2780 CSLC, and TOV21G CSLC) treated without (Control) or with CEP-1347 (300 nM for PANC-1 CSLC, 200 nM for the others) for 6 days were subjected to flow cytometric analysis of the cell-surface expression of CD133. Representative flow cytometric plots together with the percentages of CD133-positive cells are shown. **(B)** Cells treated as described in (A) were subjected to immunoblot analysis of the indicated proteins. **(C)** PANC-1 CSLC cells treated without (Control: lane 1) or with CEP-1347 (lane 2) for 6 days and those cultured in the presence of CEP-1347 for 6 days and then in its absence for the indicated days (lanes 3-5) were subjected to immunoblot analysis of the indicated proteins.

First, we treated CSCs *in vitro* with CEP-1347 at 300 nM or below, concentrations that has been shown to be clinically-relevant in human studies [11, 14–16] and confirmed not to be toxic to normal human lung fibroblasts or rat neural stem cells and also minimally toxic to CSCs (Supplementary Figure 1), to analyze the impact of the drug on the stem cell properties of CSCs. Culture of three different glioma stem cells (GS-Y01, GS-Y03, and GS-NCC01) in the presence of CEP-1347 resulted in substantial declines in the proportion of cells expressing a stem cell marker CD133 on the cell surface, which was similarly observed when other CSCs such as pancreatic (PANC-1 CSLC) and ovarian (A2780 CSLC and TOV21G CSLC) CSCs were tested (Figure 1A). To determine whether the loss of the cell surface expression of CD133 caused by CEP-1347 treatment represented loss of stem cell properties by CSCs, we examined the expression levels of other stem cell markers and also those of differentiation markers at the same time. The results indicated that CEP-1347 treatment reduced the expression levels of stem cell markers such as Sox2 and Bmi1 in all CSCs examined while inducing the expression of respective differentiation markers such as glial fibrillary acidic protein (GFAP) in glioma stem cells and E-cadherin in ovarian CSCs (Figure 1B). Increase in E-cadherin expression, however, was not observed in PANC-1 CSLC cells at least when the cells were treated with CEP-1347 for up to 6 days. To continuously monitor, therefore, the impact of CEP-1347 on E-cadherin expression in PANC-1 CSLC cells beyond 6 days, we cultured the cells in the absence of CEP-1347 thereafter because long-term exposure to CEP-1347 substantially reduced their viability. Strikingly, we found that E-cadherin expression progressively increased accompanied by constant decrease in Sox2 expression even in the absence of CEP-1347 (Figure 1C). The results, while providing evidence that CEP-1347 promotes differentiation also in PANC-1 CSLC cells, suggested that transient exposure to CEP-1347 may be sufficient to commit CSCs to differentiation into non-CSCs.

### CEP-1347 inhibits the self-renewal and tumor-initiating capacities of CSCs *in vitro*

**Figure 2:**
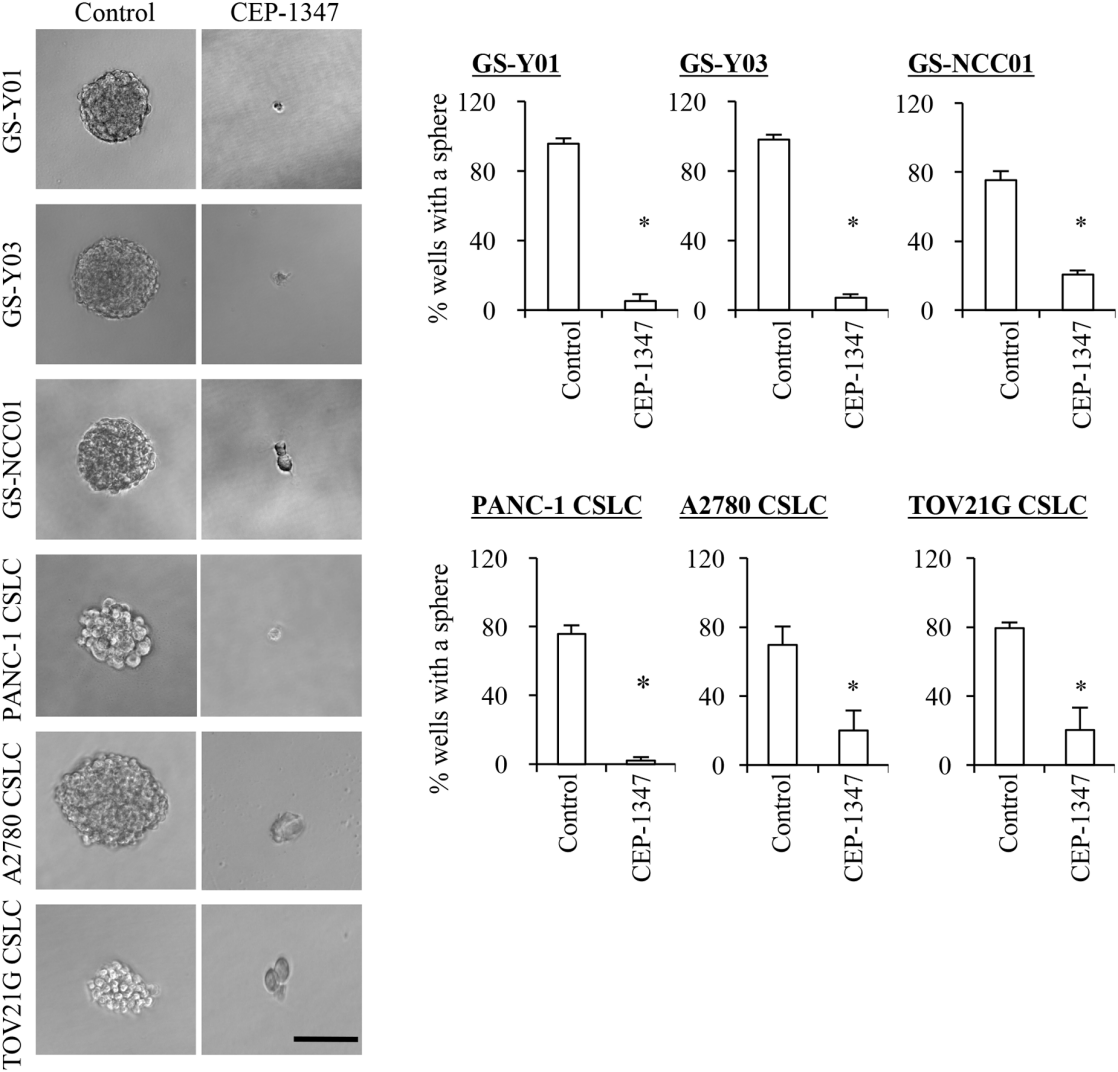
CEP-1347 treatment deprives cancer stem cells of their sphere forming ability Cells treated without (Control) or with CEP-1347 (300 nM for PANC-1 CSLC, 200 nM for the others) for 6 days were subjected to a sphere formation assay in the absence of CEP-1347. Left panels show the photographs of the representative wells. The graphs show the percentage of wells in which a tumor sphere was formed from a single viable cell. Values in the graphs represent the mean + SD from three independent experiments. ^*^P < 0.05. Bar: 100 μm.

**Figure 3:**
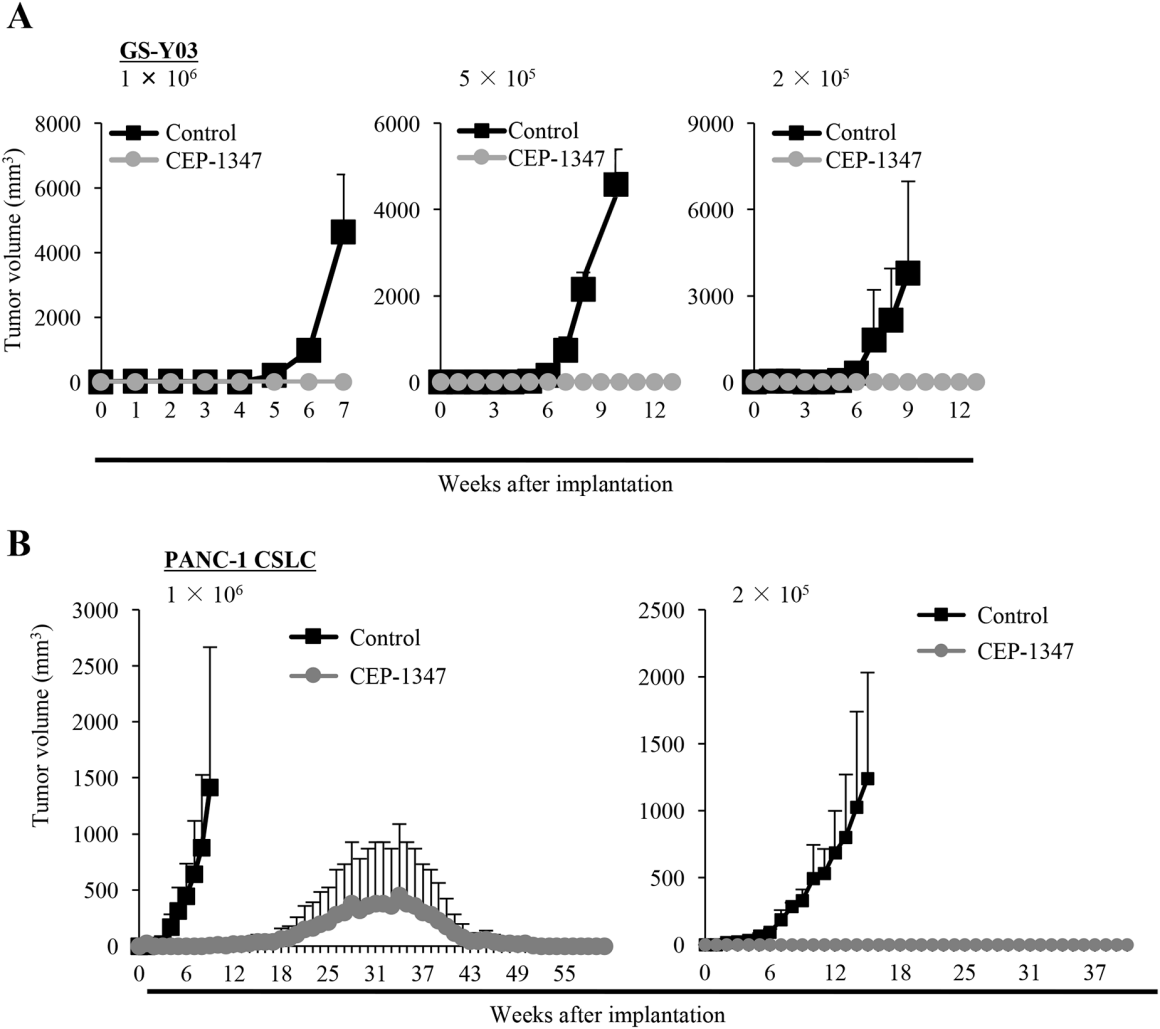
CEP-1347 inhibits the tumor-initiating capacity of cancer stem cells **(A, B)** Mice (3 for each group) were implanted subcutaneously with the indicated number of viable GS-Y03 (A) or PANC-1 CSLC (B) that had been treated with or without CEP-1347 (200 nM for GS-Y03, 300 nM for PANC-1 CSLC) for 6 days. The tumor volume was measured at the indicated time points, and results are presented as the means + SD in the graphs.

Having demonstrated that CEP-1347 is a potent driver of CSC differentiation, we next determined the effect of CEP-1347 treatment on the self-renewal and tumor-initiating capacities of CSCs by sphere formation and xenograft assays, respectively. When CSCs were cultured in the sphere-forming condition in the absence of CEP-1347 after being treated in its presence for 6 days, the number of spheres formed was significantly reduced by the preceding CEP-1347 treatment, suggesting that the CEP-1347 pretreatment had impaired their self-renewal capacity by the time they were subjected to the sphere formation assay (Figure 2). Similarly, when CSCs treated for 6 days with CEP-1347 were implanted into mice, the cells failed to form tumors with the exception of one case, whereas control-treated CSCs invariably gave rise to tumors that grew progressively (Figure 3). Notably, in a mouse implanted with CEP-1347-treated PANC-1 CSLC cells, a large (>1,000 mm^3^) tumor was formed, which nonetheless regressed spontaneously (Figure 3B, *Left*). This observation suggested the possibility that CEP-1347 impaired the ability of CSCs to perpetuate tumor growth without interfering with their engraftment in mice (see Discussion). Together, the results indicated that CEP-1347 effectively inhibits the self-renewal and tumor-initiating capacities of CSCs *in vitro*.

### Systemically administered CEP-1347 inhibits CSCs *in vivo*

**Figure 4:**
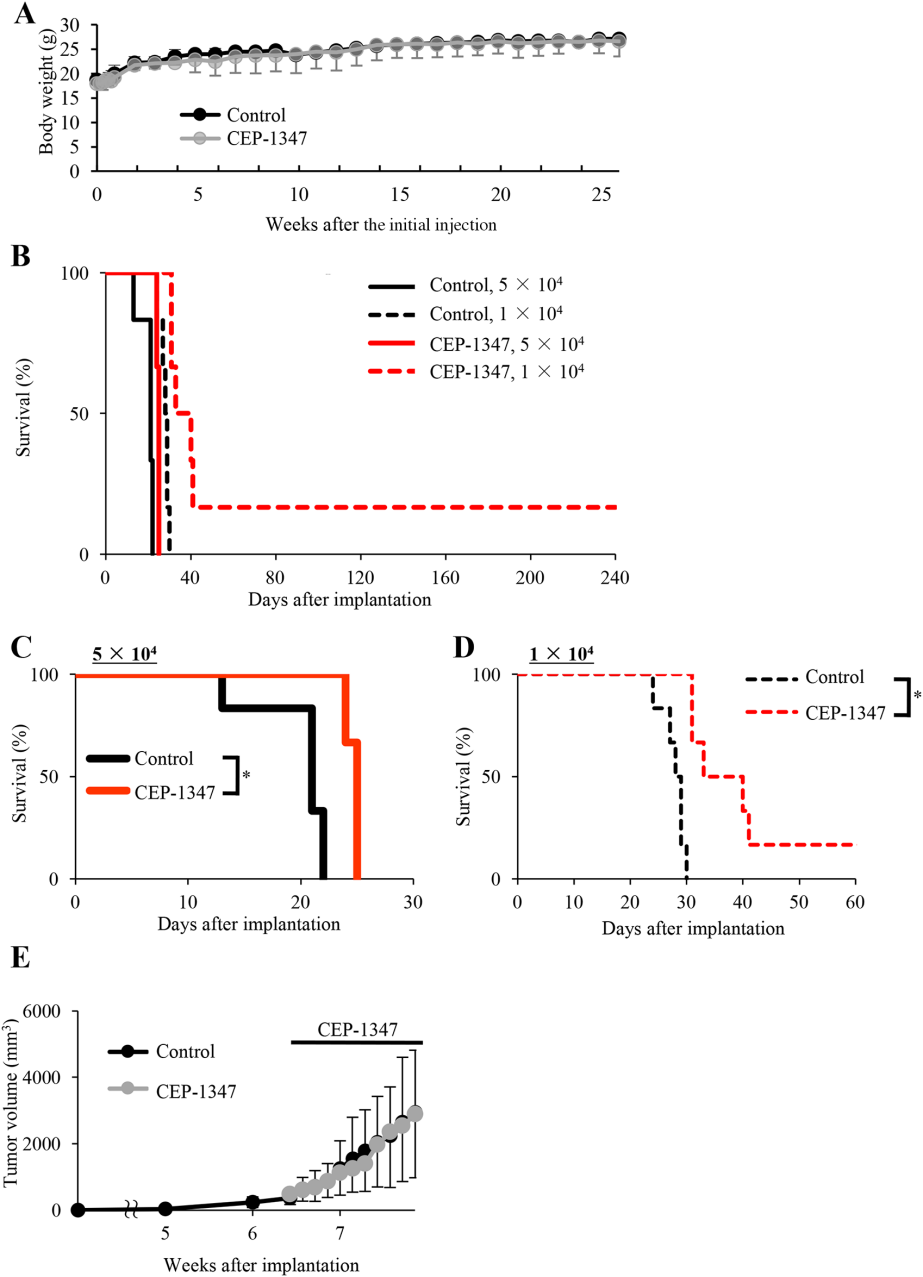
Systemically administered CEP-1347 selectively targets and inhibits tumor-initiating cancer stem cells within tumors in tumor-bearing mice **(A)** Two groups of mice (2 mice per group) were given daily intraperitoneal CEP-1347 injections (1.5 mg/kg/day) for 10 consecutive days, and their body weight was monitored at the indicated time points. Values in the graph represent the mean ± SD of the 2 mice in each group. **(B-E)** Mice implanted subcutaneously with GS-Y03 (1 × 10^6^ cells) were randomized into 2 treatment groups (4 mice per group) 6 weeks after implantation, when the average primary tumor volume reached approximately 400 mm^3^, and received a daily intraperitoneal injection of the control vehicle or CEP-1347 (1.5 mg/kg/day) for 10 consecutive days. One day after the final drug treatment, the subcutaneous tumors were excised and dissociated, and the serial dilutions of the dissociated tumor cells were transplanted intracranially into new mice, which were then observed without any further treatment. Kaplan-Meier survival curves of the mice (n = 6 for each group) are shown (B). For clarity, the survival curves of mice transplanted with 5 ×10^4^ and 1×10^4^ cells are shown separately in (C) and (D), respectively (^*^P < 0.05). The volume of the primary tumors treated without (Control) or with CEP-1347 was assessed at the indicated time points and is presented in the graph as the mean ± SD (E).

Encouraged by the potent CSC-inhibitory activity of CEP-1347 demonstrated *in vitro*, we went on to determine *in vivo* whether systemically administered CEP-1347 can target and inhibit CSCs *in situ*, *i.e.*, in tumors in animal models of human cancer. Previous clinical studies in humans demonstrated that CEP-1347 up to 100 mg/day (50 mg, twice a day orally) was well tolerated and sufficient to raise the plasma concentration of CEP-1347 to submillimolar levels [14, 16]. Since a dose of 100 mg/day in adult humans translates to ∼20 mg/kg/day in mice based on the Km values of human and mouse [17], we chose a starting dose of 1.5 mg/kg/day of CEP-1347, which is less than 1/10 the above dose and has indeed been administered successfully to mice for 1 week via the intraperitoneal route [18]. After confirming that intraperitoneal administration of 1.5 mg/kg/day CEP-1347 for 10 consecutive days does not impair the general health status of mice as monitored by their body weight (Figure 4A), we conducted a serial transplantation assay to evaluate whether systemic CEP-1347 reduces the tumor-initiating CSC population within xenograft tumors in tumor-bearing mice. To this end, we treated mice bearing subcutaneous tumors formed by the implantation of glioma stem cells according to the above treatment schedule (intraperitoneal injection of 1.5 mg/kg CEP-1347 once a day for 10 days). On the next day of the final drug administration, the subcutaneous tumors were excised and, after dissociation, tumor cells were transplanted orthotopically into the brains of new mice, which were then observed thereafter without any additional treatment. The results of the serial transplantation assay indicated that transplantation of tumor cells (5 × 10^4^ and 1 × 10^4^) derived from control-treated primary tumors invariably leads to brain tumor formation and subsequent mortality, though the survival time varied depending on the number of transplanted tumor cells, *i.e.*, reflecting the number of CSCs transplanted (Figure 4B). The results also indicated that transplantation of tumor cells (5 × 10^4^) derived from CEP-1347-treated primary tumors eventually resulted in brain tumor formation in all recipient mice. However, survival was significantly extended compared with that of the corresponding control, consistent with the idea that CEP-1347 treatment reduced tumor initiating CSCs within the primary tumors (Figure 4B and 4C). Indeed, this idea was corroborated by the observation that mice transplanted with 1 × 10^4^ tumor cells derived from CEP-1347-treated primary tumors survived significantly longer than the corresponding control mice, with one of them even surviving longer than 240 days without any signs of brain tumor formation (Figure 4B and 4D). Notably, the identical CEP-1347 treatment schedule apparently failed to inhibit the growth of the primary tumors, which is considered to be driven mainly by the proliferation of non-CSCs (Figure 4E). Collectively, these results suggested that CEP-1347 selectively inhibits tumor-initiating cells *in vivo*, preferentially targeting CSCs over non-CSCs.

**Figure 5:**
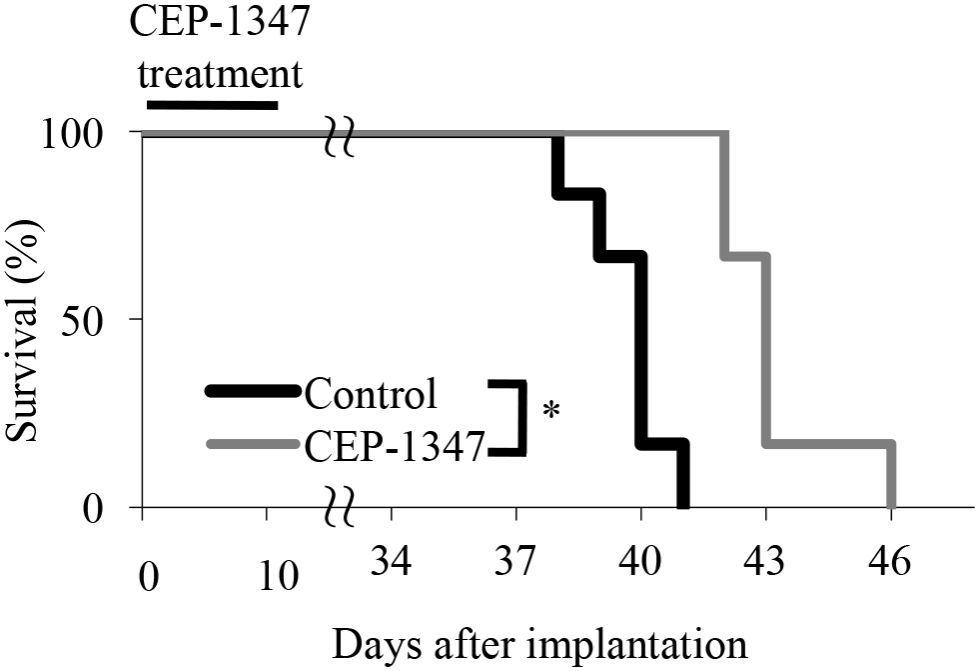
Short-term systemic CEP-1347 treatment inhibits tumor formation by glioma stem cells in an orthotopic brain tumor model Mice (n = 6 for each group) implanted intracranially with GS-Y03 cells (1 × 10^4^) underwent a daily intraperitoneal injection of the control vehicle or CEP-1347 (1.5 mg/kg/day) for 10 consecutive days, which started on the next day of intracranial implantation. Survival of mice was evaluated by Kaplan-Meier analysis, and the survival curves are shown. ^*^P < 0.05.

Given that the low dose of CEP-1347 was sufficient to reduce glioma stem cells in subcutaneous tumors significantly, we next sought to determine whether the same treatment schedule was effective in inhibiting tumor-initiation by glioma stem cells in their orthotopic location, *i.e.*, in the brain parenchyma. To this end, we stereotactically implanted glioma stem cells into mouse brain and initiated CEP-1347 treatment (intraperitoneal injection of 1.5 mg/kg CEP-1347 once a day for 10 consecutive days) on the next day of implantation. Consistent with the results of the serial transplantation assay, the identical CEP-1347 treatment schedule was similarly effective in this brain tumor xenograft model, significantly extending the survival of mice compared to control treatment (Figure 5). Thus, the results suggested that CEP-1347 can therapeutically target glioma stem cells in the brain, in line with its reported ability to penetrate the blood-brain barrier [19].

## DISCUSSION

In an attempt to identify novel CSC-targeting agents from existing drugs through drug repositioning, we conducted in this study a screening of candidate drugs and successfully uncovered the CSC-inhibitory activity of CEP-1347. *In vitro*, CEP-1347 efficiently induced differentiation of CSCs into non-CSCs at clinically-relevant concentrations, and thus inhibited their self-renewal and tumor-initiating capacities. *In vivo*, systemic administration of a low dose of CEP-1347 effectively reduced the tumor-initiating population within established tumors in tumor-bearing mice. Furthermore, the same low dose of systemic CEP-1347 provided a significant survival benefit in an orthotopic model of brain tumor. Taking into consideration the strengths of this drug described below, our data suggest that CEP-1347 may list among the most promising anti-CSC drug candidates.

Since the gold standard measure of a stem cell, operationally, is maintenance of long-term clonal growth in functional repopulation assays, carrying out secondary tumor formation assays *in vivo* using tumors initiated by a single CSC is currently considered to be the only definitive method to assess the self-renewal potential of CSCs [20, 21]. However, many CSC studies so far have employed surrogate *in vitro* assays without conducting clonal serial *in vivo* repopulation assays, which has generated much confusion in the CSC field [20]. Indeed, *in vivo* secondary tumor formation assay is rarely conducted to evaluate the CSC-targeting activity of a candidate drug, leaving its efficacy as an anti-CSC drug obscure and thus making it much less likely to succeed in future clinical trials. In this regard, the results of the serial transplantation assay conducted in this study provided evidence in support of the idea that CEP-1347 reduces cells capable of long-term clonal growth *in vivo*. Although it remains to be formally shown whether, *in vivo*, CEP-1347 reduces CSCs by killing or by depriving them of their self-renewal capacity without impairing viability, our *in vitro* data suggest that the latter possibility is more likely, with CEP-1347 driving the differentiation of CSCs into cells without self-renewal capacity *in vivo* as well as *in vitro*. Remarkably, the dose of CEP-1347 required to have a significant effect in the serial transplantation assay (and in the orthotopic treatment model) conducted in mice was, when translated to a human equivalent, less than 1/10 of the dose shown to be safe and well tolerated in human clinical trials. The demonstration of the CSC-inhibitory activity of CEP-1347 *in vivo* with much room left for dose escalation further makes this drug appealing as a candidate for clinical application.

Another strength of CEP-1347 as a candidate anti-CSC drug is that its safety profile in humans is already known, since the drug was identified through drug repositioning. Previously, based on a series of *in vitro* cell culture and *in vivo* animal studies demonstrating the efficacy of CEP-1347 in Parkinson’s disease (PD) models, CEP-1347 was advanced to evaluation in human clinical trials [22]. Following small scale, short-term clinical studies that showed twice-daily administration of 10 to 50 mg CEP-1347 resulted with safety and tolerability in plasma levels of 20 to 200 ng/mL (concentrations associated with efficacy in preclinical models of PD), a large scale phase 2/3 study was conducted to determine whether CEP-1347 would slow the progression of early PD and continue to be safe and well tolerated [11, 14, 15]. Although, unfortunately, CEP-1347 proved to be ineffective in the treatment of early PD, the results of this clinical trial demonstrated that chronic CEP-1347 treatment (10 to 50 mg, twice daily) for up to 24 months was generally safe and well tolerated. More recently, based on the beneficial effects of CEP-1347 demonstrated in *in vitro* models of HIV neurotoxicity and also in an animal model of HIV encephalitis [18, 23], a pharmacokinetic study was conducted, in which HIV patients received 50 mg CEP-1347 twice daily for 3 weeks. The results of this clinical study showed that all patients tolerated study medications well and that the mean maximal plasma CEP-1347 concentration reached 427.4 ng/mL (approximately 700 nM). Together, these data from human studies indicate that the concentration and dose required for CEP-1347 to show efficacy against CSCs *in vitro* and *in vivo* are clinically achievable, corroborating the feasibility of CEP-1347 treatment to target CSCs in humans.

While the results of this study, together with the above discussion, clearly suggest that CEP-1347 is promising as an anti-CSC drug, the molecular mechanism by which it inhibits CSCs still remains somewhat unclear. CEP-1347 was synthesized as a derivative of the natural product K-252a found in broths of *Narcodiopsis* bacterium [24]. Following the discovery that CEP-1347 is an inhibitor of the JNK signaling pathway [25], MLKs, known to act as upstream regulators of JNK, were identified as its direct targets [12]. Among the three MLKs (MLK1, MLK2, and MLK3), MLK3 was shown to be inhibited most potently by CEP-1347 [12], and CEP-1347 has since been used mainly as a MLK3 inhibitor [23, 26, 27]. In light of this known mechanism of action for CEP-1347, we have conducted pilot experiments so far to probe the role of MLK3 and JNK signaling in the inhibitory effects of CEP-1347 on CSCs. The results indicate that, whereas the JNK pathway activity in CSCs is inhibited in the presence of CEP-1347 suggesting that JNK is at least in part involved in the inhibitory effects of CEP-1347 on CSCs (Supplementary Figure 2), the effects of MLK3 knockdown on parameters such as the expression of stem cell and differentiation markers and the JNK pathway activity in CSCs failed to fully recapitulate those of CEP-1347 (M.O. and C.K., unpublished data). Thus, the findings appeared to suggest that CEP-1347 targets not only MLK3 but also the other MLKs to inhibit JNK activity and consequently the stem cell properties of CSCs. In this respect, it would be intriguing to note that MLK4, another member of the MLK family, has been recently shown to be required for the maintenance of self renewal and tumorigenicity of mesenchymal glioma stem cells [28, 29]. Although, thus, much remains to be clarified as to the contribution of each MLK to the maintenance of CSCs, delineation of their roles in CSC maintenance might provide useful information to develop CEP-1347 derivatives with further improved efficacy in the future.

In understanding how CEP-1347 inhibits the tumor-initiating capacity of CSCs, the observation that a large tumor formed by the implantation of CEP-1347-treated PANC-1 CSLC cells underwent spontaneous regression may be worth discussion. Since the spontaneous regression occurred in a single tumor, this alone would have made it impossible to exclude the possibility that it happened just by chance. However, similar phenomena have been repeatedly observed with PANC-1 CSLC cells treated with JNK inhibitors. In a serial transplantation assay, cells transplanted from primary tumors established by implanting PANC-1 CSLC cells and treated systemically with a JNK inhibitor SP600125 formed secondary tumors that eventually regressed without any treatment [30]. Similarly, implantation of PANC-1 CSLC cells pretreated with another JNK inhibitor AS602801 *in vitro* resulted in the formation of tumors that regressed spontaneously [7]. Combined, these findings strongly suggest that JNK inhibition in PANC-1 CSLC cells may lead not only to ‘complete loss’ of self renewal but also to ‘limited’ self renewal in some cells, allowing the formation of tumors that fail to grow continuously. Importantly, our observation that PANC-1 CSLC cells treated with CEP-1347 *in vitro* formed spontaneously regressing tumors has two significant implications: 1) CEP-1347’s effects on human CSCs are not dependent on microenvironmental interaction between the cells and the host (animals or humans), 2) CEP-1347 selectively targets a cell-intrinsic mechanism(s) essential for the perpetuation of tumor growth but not for cells’ engraftment in mice, an artificial process unique to mouse xenograft models. Such properties of CEP-1347 would work in favor of increasing the probability that CEP-1347 will successfully inhibit tumor initiation from CSCs not only in xenograft models but also in humans.

In conclusion, we have demonstrated in this study CEP-1347 promotes CSC differentiation at clinically-relevant concentrations *in vitro* and as such effectively inhibits tumor formation by CSCs *in vivo*. Although the exact mechanism behind the CSC-inhibitory activity of CEP-1347 and the role of MLKs therein remain to be elucidated, our findings, together with the solid safety records of the drug in humans, suggest that adding CEP-1347 to current cancer treatment regimens is a rational and feasible approach to preventing post-treatment recurrence and/or metastasis from CSCs surviving treatment, in particular from those dependent on JNK for their stem cell state.

## MATERIALS AND METHODS

### Reagents and antibodies

CEP-1347 was purchased from TOCRIS Bioscience (Bristol, UK) and was dissolved in DMSO to prepare a 1 mM stock solution. Anti-CD133 (W6B3C1) was from Miltenyi Biotech (Bergisch Gladbach, Germany). Antibodies against Sox2 (#3579), Bmi1 (#6964), GFAP (#3670), and GAPDH (#5174) were purchased from Cell Signaling Technology Inc. (Beverly, MA, USA), except that the antibodies used for the detection of Sox2 (MAB2018) and Bmi1 (05-637) in glioma stem cells were purchased from R&D Systems Inc. (Minneapolis, MN, USA) and Millipore (Billerica, MA, USA), respectively. Ant-E-cadherin (sc-8426) was from Santa Cruz Biotechnology, Inc. (Santa Cruz, CA, USA). Horseradish peroxidase (HRP)-conjugated secondary antibodies and Alexa Fluor^®^ 488-conjugated secondary antibody were purchased from Jackson ImmunoResearch Laboratories Inc. (West Grove, PA, USA) and Thermo Fisher Scientific, (Waltham, MA, USA), respectively.

### Cell culture

The establishment and characterization of the human CSCs used in this study (GS-Y01, GS-Y03, GS-NCC01, PANC-1 CSLC, A2780 CSLC) has been described [30–32]. A subline of TOV21G human ovarian cancer cell line with cancer stem-like properties (TOV21G CSLC) was established from TOV21G according to the method described previously [30]. These CSCs and rat neural stem cells (R&D Systems Inc.) were maintained under the monolayer stem cell culture condition [30–33]. Briefly, cells were cultured on collagen-I-coated dishes (IWAKI, Tokyo, Japan) in the stem cell culture medium (DMEM/F12 medium supplemented with 1% B27 [Thermo Fisher Scientific], 20 ng/mL EGF and FGF2 [Peprotech Inc., Rocky Hill, NJ, USA], D-(+)-glucose [final concentration, 26.2 mM], L-glutamine [final concentration, 4.5 mM], 100 units/mL penicillin, and 100 μg/mL streptomycin). The stem cell culture medium was changed every 3 days, and EGF and FGF2 were added to the culture medium every day. Normal human IMR90 fetal lung fibroblasts were obtained from ATCC and maintained in DMEM supplemented with 10% FBS, 100 U/mL penicillin, and 100 μg/mL streptomycin. The authenticity of PANC-1 CSLCs and A2780 CSLCs was verified by genotyping of short tandem repeat loci (Bio-Synthesis Inc., Lewisville, TX, USA) followed by comparison to the ATCC STR database (http://www.atcc.org/STR_Database.aspx) for Human Cell Lines. All IMR90 and rat neural stem cell experiments were performed using low passage number (< 8) cells. Throughout the study, the cell number was determined using a hemocytometer, and the cell viability was examined by the dye exclusion method (0.2% trypan blue). Cell viability (%) was defined as 100 × ‘the number of viable cells’/ (‘the number of viable cells’ + ‘the number of dead cells’).

### Flow cytometric analysis

Flow cytometric analysis was conducted as previously described [7, 30, 34]. Dissociated cells were washed with ice-cold phosphate-buffered saline (PBS), fixed with 4% (w/v) paraformaldehyde for 10 min at room temperature (RT), and washed twice with PBS. The cells were blocked in FCM buffer (0.5% [w/v] bovine serum albumin, 0.1% [w/v] NaN_3_ in PBS) for 1 h, followed by PBS rinses and further incubation with anti-CD133 antibody in the FCM buffer overnight at 4°C and then with the Alexa Fluor^®^ 488 goat anti-mouse IgG for another 1 h at RT. Gating for viable single cells was established using forward scatter in the isotype control samples. The isotype control samples were used to establish a gate in the fluorescein isothiocyanate channel. Cells showing a signal for CD133 above the gate established by the isotype control were deemed CD133-positive. All flow cytometric experiments were run on FACSCanto™ II Flow Cytometer (BD Biosciences, Franklin Lakes, NJ, USA) and the data were analyzed using FlowJo software, version 7.6.5 (Tree star Inc., Ashland, OR, USA).

### Immunoblot analysis

Immunoblot analysis was conducted essentially as previously described [7, 34, 35]. In brief, cells were washed with ice-cold PBS and lysed in an appropritate buffer. Glioma stem cells were lysed in RIPA buffer (10 mM Tris-HCl [pH 7.4], 0.1% SDS, 0.1% sodium deoxycholate, 1% NP-40, 150 mM NaCl, 1 mM EDTA, 1.5 mM Na_3_VO_4_, 10 mM NaF, 10 mM sodium pyrophosphate, 10 mM sodium β-glycerophosphate and 1% protease inhibitor cocktail set [Sigma]). To solubilize membrane proteins, the other CSC lines were lysed in a 1:1 mixture of RIPA and Laemmli buffer (125 mM Tris-HCl [pH 6.8], 20% glycerol, 4% SDS). After centrifugation for 10 min at 14,000 × g at 4°C, the supernatants were recovered as cell lysates, and the protein concentration of the cell lysates was determined by a BCA protein assay kit (Pierce Biotechnology Inc., Rockford, IL, USA). Cell lysates containing equal amounts of protein were separated by SDS-PAGE and transferred to a polyvinylidene difluoride membrane. The membrane was probed with a primary antibody and then with an appropriate HRP-conjugated secondary antibody according to the protocol recommended by the manufacturer of each antibody. Immunoreactive bands were visualized using Immobilon Western Chemiluminescent HRP Substrate (Millipore) and detected by ChemiDoc™ Touch Imaging System (Bio-Rad Laboratories, Hercules, CA, USA).

### Sphere formation assay

The sphere formation assay was performed as previously described [30–32]. After dissociation, single cells were serially diluted in the stem cell culture medium and seeded into non-coated 96-well plates such that each well contained a single cell. Wells containing a single viable cell were marked under a phase-contrast microscope on the next day, and 7 days after seeding, the percentage of marked wells with a sphere relative to the total number of marked wells was determined.

### Mouse studies

Mouse xenograft studies were conducted essentially as previously described [8, 30, 31]. For subcutaneous implantation, 6- to 9-week-old male BALB/cAJcl-nu/nu mice (CLEA Japan Inc., Tokyo, Japan) were implanted subcutaneously in the flank region with cells suspended in 200 μL of sterilized PBS under avertin (0.375 g/kg intraperitoneally) anesthesia. For intracranial implantation, mice were anesthetized with avertin before cells suspended in 10 μL of PBS were injected stereotactically in the left corpus striatum (2.5 mm anterior and 2.5 mm lateral to the bregma, and 3.0 mm deep) of 6-week-old male BALB/cAJcl-nu/nu mice. In principle, the general health status of the recipient mice was monitored on a daily basis after implantation. The volume of subcutaneous tumors was determined on a weekly basis by measuring tumor diameters (measurement of 2 perpendicular axes of tumors) using a caliper and calculated as 1/2 × (larger diameter) × (smaller diameter)^2^.

For serial transplantation, primary tumors treated as described in the figure legend were excised, and, after a wash in chilled sterile PBS, were transferred into DMEM/F12, minced with scissors, and incubated in TrypLE™ Express (Thermo Fisher Scientific) for 30 min at 37°C. After being rinsed with DMEM/F12, the cells were resuspended in DMEM/F12 and filtered through a 70-μm strainer. The single cell suspension was then intracranially injected after cell number and viability were determined. For systemic administration of CEP-1347, the CEP-1347 stock solution (1 mM in DMSO) was diluted in PBS to prepare 200 μL solutions for each injection. The CEP-1347 solutions were injected intraperitoneally into nude mice. All control- and CEP-1347-treated mice received an equal volume of DMSO per body weight (3.6 mL/kg). All animal experiments were performed under a protocol approved by the Animal Research Committee of Yamagata University.

### Statistical analysis

Results are expressed as the mean ± standard deviation (SD), and differences were compared using the two-tailed Student’s *t*-test. Mouse survival was evaluated by the Kaplan-Meier method and analyzed by using the log-rank test. *P*-values < 0.05 were considered statistically significant and are indicated with asterisks in the figures.

## SUPPLEMENTARY MATERIALS FIGURES


