# Supplementary material for: Repositioning CEP-1347, a chemical agent originally developed for the treatment of Parkinson’s disease, as an anti-cancer stem cell drug
Source: Oncotarget. 2017 Oct 24;8(55):94872–82. doi: 10.18632/oncotarget.22033 (PMC5706919; doi:10.18632/oncotarget.22033)
Supplement: Supplementary file 1 [file oncotarget-08-94872-s001.pdf]

Repositioning CEP-1347, a chemical agent originally developed for the treatment of Parkinson's disease, as an anti-cancer stem cell drug

SUPPLEMENTARY MATERIALS

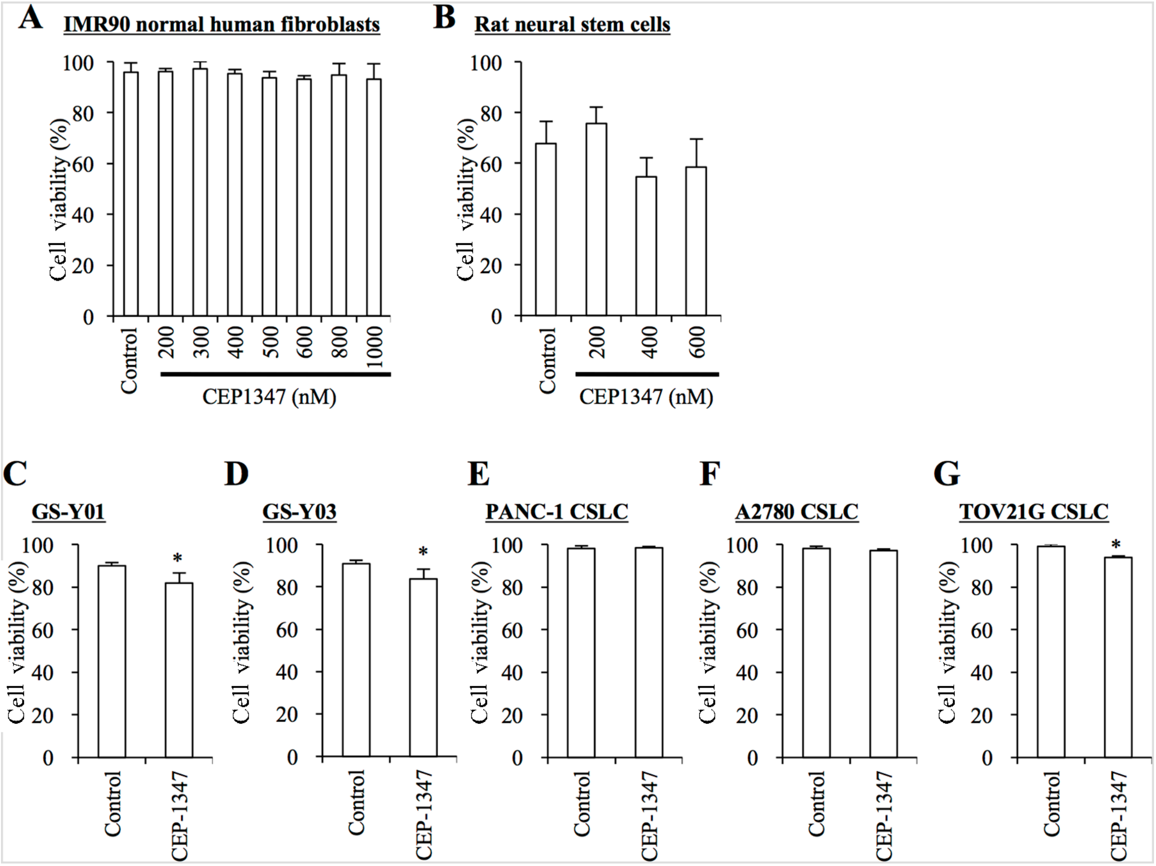

Supplementary Figure 1: Effect of CEP-1347 on the viability of normal fibroblasts, neural stem cells, and cancer stem cells. IMR90 normal human fibroblasts **(A)**, rat neural stem cells **(B)**, GS-Y01 **(C)**, GS-Y03 **(D)**, PANC-1 CSLC **(E)**, A2780 CSLC **(F)**, and TOV21G CSLC **(G)** treated without (Control) or with CEP-1347 at the indicated concentrations (A, B), 300 nM (PANC-1 CSLC), and 200 nM (the other cancer stem cells) for 6 days were subjected to cell viability assay using trypan blue. The values in the graphs represent means + SD from triplicate samples of a representative experiment repeated with similar results.

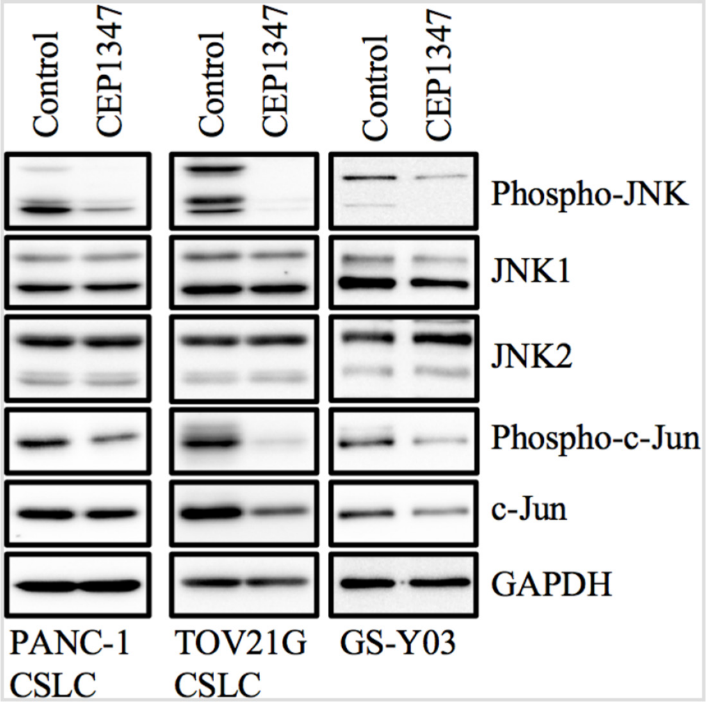

Supplementary Figure 2: Effect of CEP-1347 on the JNK pathway in cancer stem cells. Cells treated without (Control) or with CEP-1347 (300 nM for PANC-1 CSLC, 200 nM for the others) for 24 h (PANC-1 CSLC and TOV21G CSLC) or 4 h (GS-Y03) were subjected to immunoblot analysis of the indicated proteins.
